# Design and Theoretical Analysis of a Resonant Sensor for Liquid Density Measurement

**DOI:** 10.3390/s120607905

**Published:** 2012-06-08

**Authors:** Dezhi Zheng, Jiying Shi, Shangchun Fan

**Affiliations:** School of Instrument Science and Opto-Electronics Engineering, New Main Building B504, Beijing University of Aeronautics and Astronautics, Beijing 100191, China; E-Mails: zhengdezhi@buaa.edu.cn (D.Z.); shangcfan@buaa.edu.cn (S.F.)

**Keywords:** liquid density measurement, resonant sensor, tuning fork, natural frequency

## Abstract

In order to increase the accuracy of on-line liquid density measurements, a sensor equipped with a tuning fork as the resonant sensitive component is designed in this paper. It is a quasi-digital sensor with simple structure and high precision. The sensor is based on resonance theory and composed of a sensitive unit and a closed-loop control unit, where the sensitive unit consists of the actuator, the resonant tuning fork and the detector and the closed-loop control unit comprises precondition circuit, digital signal processing and control unit, analog-to-digital converter and digital-to-analog converter. An approximate parameters model of the tuning fork is established and the impact of liquid density, position of the tuning fork, temperature and structural parameters on the natural frequency of the tuning fork are also analyzed. On this basis, a tuning fork liquid density measurement sensor is developed. In addition, experimental testing on the sensor has been carried out on standard calibration facilities under constant 20 °C, and the sensor coefficients are calibrated. The experimental results show that the repeatability error is about 0.03% and the accuracy is about 0.4 kg/m^3^. The results also confirm that the method to increase the accuracy of liquid density measurement is feasible.

## 1. Introduction

Density is one of the characteristic physical properties of a substance, which can be used to understand other physical and chemical properties, for example, the isothermal compressibility coefficient or the thermal expansion coefficient [1].

The on-line density sensor was developed initially for monitoring the retrieval operations from the radioactive waste storage tanks at the Hanford Site in Eastern Washington State (USA), and it is also of interest for many other applications [2,3]. The sensor can be used in a pipeline for process control in the petrochemical industry, in the production of chemical reagents, in food processing, in the production of paper, in the production of textiles, and so on. The sensor can also be placed in a vat to determine the density of the contents [4]. At present, the study on the liquid density measurement abroad is focused on ultrasonic method, but the ultrasonic measurement devices can only reach accuracies of 0.1% [5], which are still far from those achievable with laboratory measurement instruments that can reach 0.01% or better. Therefore, more accurate measurement sensors must be developed [6].

A liquid density measurement sensor with the tuning fork as the sensitive component, which is based on the resonance principle, is described in this paper and developed to measure density of liquids, static or in motion. It can measure liquid density directly, being a quasi-digital sensor, which not only has simple structure, small size, light weight, but also has high precision and reliability [7,8]. According to the dynamic principle, an approximate parameters model of the tuning fork is established, and the impact of structural parameters, temperature and liquid on the natural frequency of the tuning fork are also analyzed either theoretically or by simulation, along with experimental results that showed the better performance of the sensor to measure liquid density.

The remainder of this paper is organized as follows: theoretical analysis and simulation results are described in Section 2, the sensor fabrication, system configuration and hardware design are presented in Section 3, and Section 4 describes the experiments and results.

## 2. Theory Analysis and Simulation

### 2.1. Working Principle

The sensitive component of resonant sensor must vibrate at its natural frequency stably during operation. Discussing the infinitesimal element of sensitive component, it can be regarded as a single freedom system theoretically. The natural frequency of the system relates to the equivalent mass and the equivalent stiffness only.

The tuning fork liquid density measurement sensor places the tuning fork driven by electromagnetic or piezoelectric method in the measuring chamber, and then the actuator transmits alternating force to the tuning fork to make it vibrate in accordance with its natural frequency. When the tuning fork contacts with the liquid measured, the added mass of the tuning fork changes, and results in vibration frequency (vibration cycle) changes. The detector picks up the vibration signal to detect the vibration frequency. By measuring the changes of the natural frequency or vibration cycle, the density of the liquid tested can be determined. Therefore, it is very important to obtain the natural frequency of the tuning fork exactly to ensure the excellent performance of the sensor.

**Figure 1.**
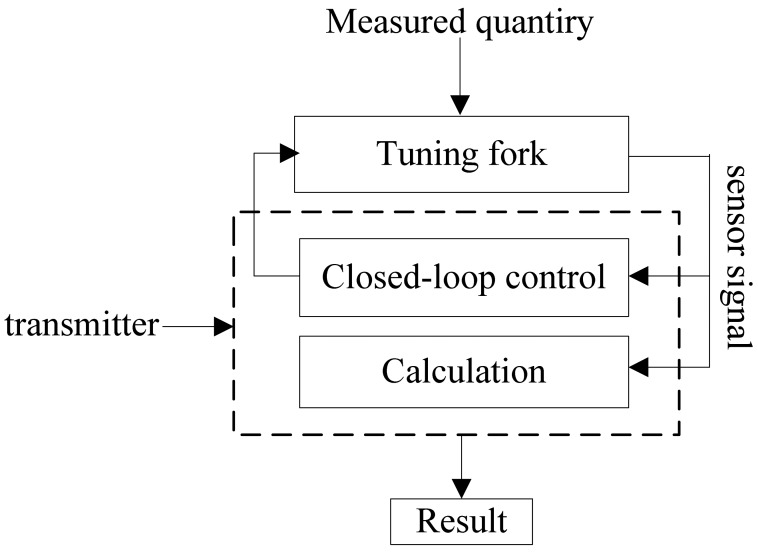
Schematic of the resonant tuning fork liquid density measurement sensor.

The sensor is composed of the sensitive unit and the closed-loop control unit, where the sensitive unit consists of the actuator, the resonant tuning fork and the detector. Resonant tuning fork senses the liquid density directly, and the detector sends the signal which carries measurement information forward to the closed-loop control unit for processing and output density value, while the closed-loop control unit output excitation signal to control the actuator and then drive the tuning fork. The basic configuration of the sensor is shown in Figure 1.

### 2.2. Resonant Frequency of the Tuning Fork

As can be seen from the analysis above, the natural frequency of the tuning fork has important impact on the performance of the sensor. In this part of the paper, an approximate parameters model of the tuning fork is established, and the impact of liquid density, position of the tuning fork, temperature and structural parameters on the natural frequency of the tuning fork are also analyzed both theoretically and by simulation.

#### 2.2.1. Resonant Frequency in the Ideal Condition

The vibration of a tuning fork can be equivalent to the vibration of a cantilever beam. The vibration frequency can be obtained from calculating Euler Equation and described as follows:

(1) $$f_{r}=\frac{{\left(\beta_{r}l\right)}^{2}}{2\pi }\sqrt{\frac{EJ}{\rho Al^{4}}},r=1,2,\cdots$$

where *ρ* is mass per unit volume, *A* is cross sectional area, *l* is the length, *EJ* is bending rigidity of the cross section, and *βl* can be calculated from the equation $\cos\beta l=-\frac{1}{\text{ch}\beta l}$. Equation (1) shows that the vibration frequency of the cantilever beam relates to the cross-sectional area and the length [9].

#### 2.2.2. Resonant Frequency Dependence on Liquid

**Figure 2.**
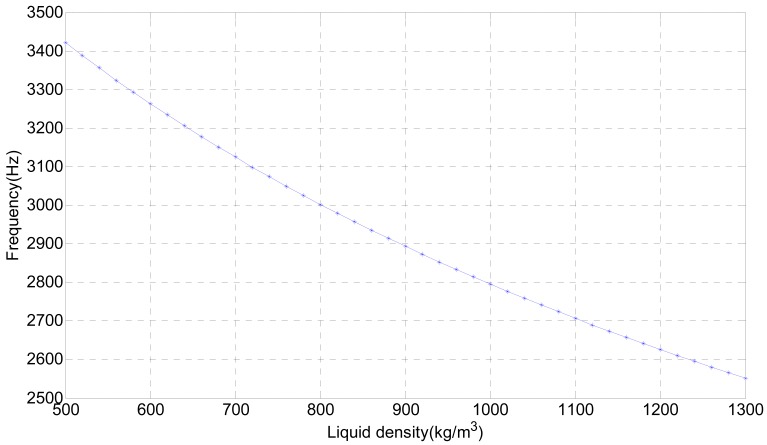
Natural frequency of the tuning fork changes with the liquid density.

Through the study on vibration of free-free beams under liquid [10], it is assumed that the liquid is ideal, incompressible and without spin, and based on the Laplace equation:

(2) $$\frac{\partial^{2}\phi }{\partial x^{2}}+\frac{\partial^{2}\phi }{\partial z^{2}}=0$$

we can get the liquid velocity potential function *φ*(*x,z,t*), and the changes of vibration frequency of a cantilever beam when the depth of the beam in the liquid changes. On this basis, we also have introduced the change of the first-order vibration frequency of the tuning fork when the depth in the liquid changes or the liquid density changes, as given by Equation (3) below:

(3) $$f'=\frac{f_{0}}{\sqrt{1+0.183\frac{4\rho 'h}{\pi \gamma }}}$$

(4) $$\rho '=\frac{({f_{0}}^{2}T^{2}-1)\pi \gamma }{0.732h}$$

where *f*_0_ is natural frequency of the tuning fork in the air, *ρ*′ is the liquid density, *T* is the vibration cycle of the tuning fork, *T* = 1/*f*′, *h* is the depth of the tuning fork into the liquid, and *γ* is density of the tuning fork per unit width. When the depth of the tuning fork in the liquid is fixed, the vibration frequency decreases as the liquid density increases. Figure 2 shows the change of natural frequency with the liquid density.

Generally speaking, the follow equation is often used to calculate the liquid density:

(5) $$\rho '=m_{0}+m_{1}T+m_{2}T^{2}$$

Where *ρ*′ is the liquid density, *_T_* is the vibration cycle of the tuning fork, *T* = 1/*f*′, *m*_0_, *m*_1_ and *m*_2_ are the sensor coefficients which can be determined through calibration experiments.

When vibrating while immersed in a liquid, the vibration frequency of the tuning fork decreases as its depth in the liquid increases. When the depth of the tuning fork in the liquid is fixed, the vibration frequency also decreases as the liquid density increases. The relation between liquid density *ρ*′ and vibration cycle of the tuning fork *T* is as indicated by Equation (5). As long as the vibration cycle of the resonant component in the liquid is measured, the density of the liquid under test can be calculated, thus enabling on-line density measurements of liquids in real-time.

#### 2.2.3. Resonant Frequency Dependence on Temperature

Temperature affects its performance if there are gradients around the sensor. The impact of temperature on the natural frequency is mainly due to the impact on the elastic modulus of the material. The relation between interaction potential energy *U*(*r*) and their distance *r* of two atoms for various types of crystals can be written as:

(6) $$U(r)=-\frac{A}{r^{n}}+\frac{B}{r^{m}}$$

where *A, B, n* and *m* are constants which are greater than zero. The first item is the attractive energy, and the second is the repulsive energy [11]. Now we choose a simple cubic crystal as model to calculate the relation between *_E_* and *T*, set the crystal under tension along the axis, so when the tension changes by d*f*, the atomic spacing *r* changes by d*r*, then the cross-sectional area *r*^2^ of unit cell is unchanged. Therefore, the elastic modulus of crystal is:

(7) $$E=\frac{\frac{\text{d}f}{r^{2}}}{\frac{\text{d}r}{r}}=\frac{\text{d}f}{r\text{d}r}$$

where $\frac{\text{d}f}{r^{2}}$ and $\frac{\text{d}r}{r}$ are the stress and strain, respectively. The binding force *f* of the crystal only relates to the first item of Equation (6), and its size is given by:

(8) $$f(r)=\frac{\text{d}U(r)}{\text{d}r}=\frac{nA}{r^{n+1}}$$

Differentiating Equation (8) with respect to *r* and substituting in Equation (7), considering *K* = −(*n* +1)*A*, *Q* = *n* +3, this leads to:

(9) $$E=\frac{nk}{r^{Q}}$$

Assuming the interatomic distance still obeys the following rules when the crystal expands by heat:

(10) $$r=r_{0}(1+\alpha T)$$

where *r*_0_ is the interatomic distance when absolute temperature *T*_0_ = 0, *α* is linear expansion coefficient of the crystal, and its differential defined type is:

(11) $$\{\begin{array}{c} \alpha =\frac{1}{r}\frac{\text{d}r}{\text{d}T}\\ \eta =-\frac{1}{r}\frac{\text{d}E}{\text{d}T} \end{array}$$

where *η* is the temperature coefficient of the elastic modulus *E*, d*E* = −*ηE*d*T* illustrates that elastic modulus decreases as the temperature increases, and the increment d*E* of elastic modulus *E* is negative. From equations above, we get:

(12) $$\{\begin{array}{c} E=E_{0}{\left(\frac{1+\alpha T}{1+\alpha T_{0}}\right)}^{-Q}\approx E_{0}(1-Q\alpha T)\\ Q=\overset{\eta }{\;}/\underset{\alpha }{\;} \end{array}$$

(13) $$E=E_{0}(1-\eta T)$$

Equation (13) shows that the elastic modulus of metallic materials decreases linearly with increasing temperature, depending on the temperature coefficient *η* of the elastic modulus. The values of *η* for metallic materials such as iron, tungsten, duralumin and carbon steel to be investigated are scant, while the experimental data of linear expansion coefficients *α* are more abundant, their experimental relation (*α*/*η*) × 10^3^ is approximately equal to 40, so we get *Q* = 25, then Equation (13) will take the form:

(14) $$E=E_{0}(1-25\alpha T)$$

Equation (14) is the general law of elastic modulus of metallic material changes with temperature. For most metallic materials, *α* changes when the temperature increases, but the small change could be considered as constant. Equation (1) will be on the form:

(15) $$f_{r}=\frac{{\left(\beta_{r}l\right)}^{2}}{2\pi }\sqrt{\frac{E_{0}(1-25\alpha T)J}{\rho Al^{4}}},r=1,2,\cdots$$

where *α* is linear expansion coefficient of the metallic materials, *T* is Kelvin, *E*_0_ is elastic modulus at absolute zero, *ρ* is density of the metallic materials, *J* is the moment of inertia, *A* is the cross sectional area, *l* is the length. Equation (14) shows the elastic modulus changes with temperature and Equation (15) verifies that the influence of temperature on the natural frequency of tuning fork is obvious, thus, a temperature sensor is required to detect the liquid measured in real-time to compensate for the changes in the elastic modulus of the tuning fork.

### 2.3. Simulation

**Figure 3.**
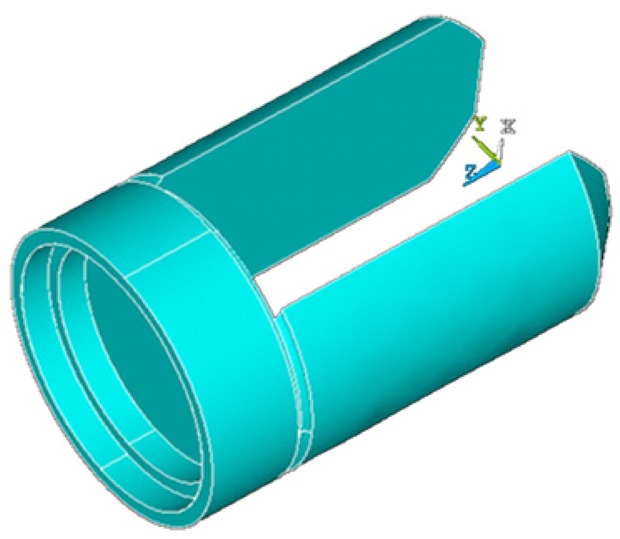
Tuning fork model.

Finite element analysis software is used to analyze the structural parameters which have an influence on the natural frequency of the tuning fork. The tuning fork oscillator is designed on this basis. The first vibrating mode is chosen as the working mode, to maintain the lowest working energy and simple mode shape. Figure 3 is the tuning fork model obtained through the finite element analysis software, and the natural frequency of the first order is 3,940.5 Hz. The natural frequency of the tuning fork based on the theoretical analysis is 3,910.82 Hz, and the measured value is 3,961.9 Hz. The finite element analysis software considers the impact of factors on the natural vibration frequency of the oscillator more comprehensively, and the matrix for calculation is more complete, therefore, the results is closer to the measured value, while Equation (1) omits a number of factors, some of which (such as constraints, *etc.*) have a great influence on the vibration frequency, so the difference between calculated value and measured value is larger, only as a reference. Since the actual processing of the harmonic oscillator, the structural parameters and material parameters are difficult to guarantee to consistent with the theoretical value, so it is understandable that there is certain difference between the theoretical result and the measured value.

According to Equation (1), the vibration frequency of the tuning fork changes as the cross-sectional area and the length change. For the tuning fork, the first-order natural frequency depends on its structural properties. In modal analysis, we analyze the impact of structure parameters on natural frequency by changing the length and width (cross-sectional area) of the tuning fork [12].

**Figure 4.**
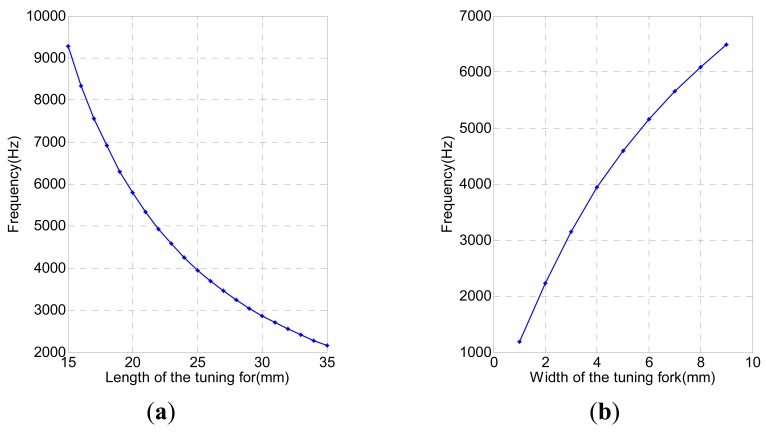
(**a**) Natural frequency of the tuning fork changes with the length; (**b**) Natural frequency of the tuning fork changes with the width.

Figure 4 shows how the first-order natural frequency of the tuning fork decreases as the length increases, and increases as the width increases. First we maintain the width invariable (4 mm) and change the length (15∼35 mm), giving the curve shown in Figure 4(a) that relates the change of natural frequency with the length. Then we maintain the length (12 mm) as a constant and change the width (1∼9 mm), and in this case the curve that shows the resulting change of natural frequency with the width is as indicated by Figure 4(b). The simulation result is identical with that of the theoretical analysis. Therefore, structural parameters must be taken into account when designing the sensor.

## 3. Sensor Fabrication and Digital Closed-Loop Control System Design

To verify the feasibility of the new design, test samples were fabricated by finish machining technology, and a digital closed-loop control system for the newly designed sensor was built up to interpret the resonant frequencies into liquid density data.

### 3.1. Tuning Fork Fabrication

**Figure 5.**
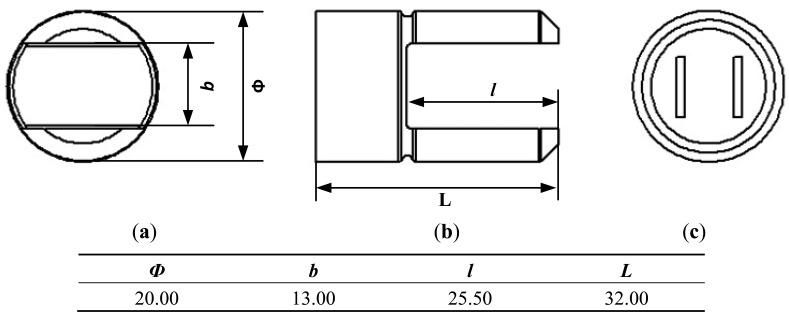
(**a**) Front view of the tuning fork; (**b**) Left view of the tuning fork; (**c**) Back view of the tuning fork (unit: mm).

Stainless steel 316 L, whose material properties are shown in Table 1, was used to make the tuning fork. The tuning fork is fabricated through finish machining technology with the specific dimensional parameters shown in Figure 5(a,b). The actuator and detector are a pair of distributed bi-piezoelectric actuators with simple structure and low power consumption. The bi-piezoelectric actuators are symmetrical, perpendicular to the tuning fork, as shown in Figure 5(c).

**Figure 6.**
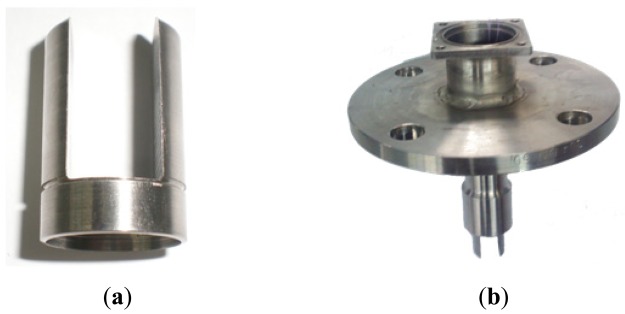
(**a**) Tuning fork; (**b**) Support flange.

The tuning fork is shown in Figure 6(a), and is supported by a flange whose stiffness is approximately infinite, shown in Figure 6(b). The flange does not vibrate, therefore, it does not affect the liquid density measurement process.

### 3.2. Digital Closed-Loop Control System

**Figure 7.**
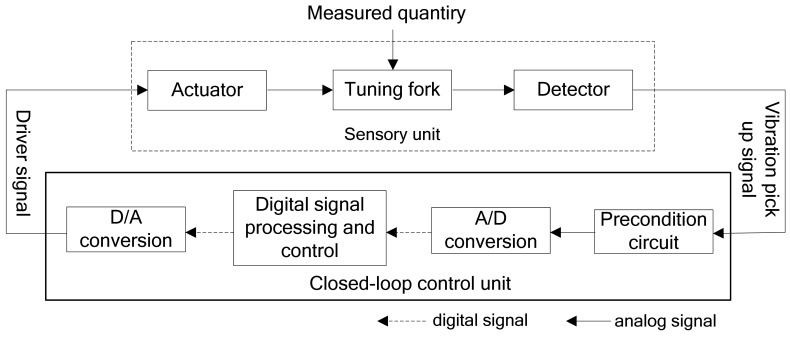
Block diagram of digital closed-loop control system.

To improve the measurement performance, high speed digital circuits and digital signal processing technology are used in the transmitters to replace conventional analog control system. The basic configuration of the closed-loop control system is illustrated in Figure 7. It mainly includes a preconditioning circuit, digital signal processing and control unit, analog-to-digital converter and digital-to-analog converter. The tuning fork senses the liquid density directly, and the detector sends the analog signal that carries measurement information to the preconditioning circuit for amplification and filtering. Then the analog-to-digital converter converts the amplified and filtered analog signal to a digital signal, and then to the closed-loop control unit to track the signal frequency, control phase difference and amplitude, and output the density value, while the closed-loop control unit outputs an excitation signal to control the actuator to drive the tuning fork vibrate in accordance with the changing vibration frequency in real-time. As seen in the configuration, to obtain the desired frequency, phase, and amplitude characteristics, a “positive feedback” technique is used herein in which a sensor signal (having the desired frequency, phase and amplitude characteristics) is fed back to the tuning fork via the drive signal.

**Figure 8.**
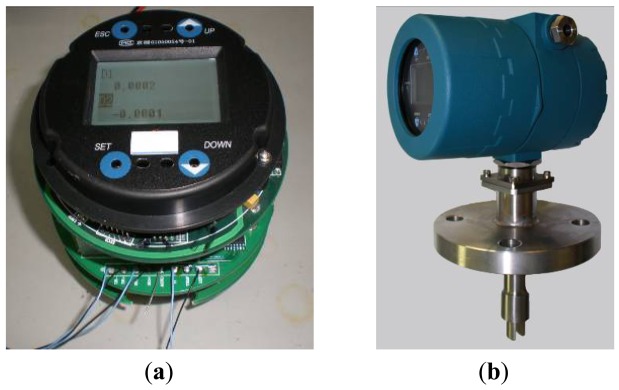
(**a**) Digital closed-loop control system; (**b**) Theoretical prototype.

Figure 8(a) shows the digital closed-loop control system, and the theoretical prototype designed to do the confirmation of function is shown in Figure 8(b).

## 4. Experiments and Discussion

### 4.1. Calibration Experiments

Calibration experiments are taken at constant 20 °C, and five different uniform densities of the liquids to be measured (alcohol if different concentration, water, CuSO_4_) were prepared. The float densitometer with the differentiate force of 1.0 kg/m^3^ is the standard density meter. Both the resonant density sensor and the standard float-type density meter are placed into the liquid to be tested, and the liquid tested is placed in the temperature controller (GDS-50L type, accuracy of ±0.1 °C) at a constant temperature of 20 °C. When the temperature is stable, the vibration cycle *T* of the resonant tuning fork and the float densitometer readings *ρ_r_* are recorded and the least square method for curve fitting used with the measured density *ρ*′ and the vibration cycle *T* of the tuning fork to get the sensor coefficients *m*_0_, *m*_1_, *m*_2_ under 20 °C which are −2,500.6776 kg/m^3^, −0.2756 kg/(m^3^·μs), 0.026778 kg/(m^3^·μs^2^) respectively, and then we can calculate the liquid density through Equation (5). Therefore, the equation for calculating the liquid density is:

(16) $$\rho '=-2500.6773-0.2756T+0.026778T^{2}$$

where *ρ*′ (unit: kg/m^3^) is the liquid density, *T* (unit: μs) is the vibration cycle of the tuning fork. Results of the experiments are shown in Table 2. They confirm that the repeatability error of the sensor is about 0.03% and the accuracy is about 0.4 kg/m^3^.

### 4.2. Temperature Experiments

**Figure 9.**
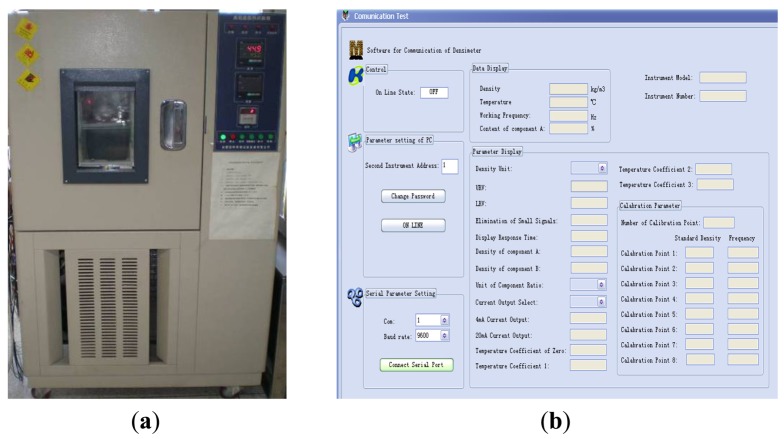
(**a**) Temperature controller(GDS-50L); (**b**) Communication software.

Temperature experiments were performed to check the impact of this factor on the natural frequency. The temperature environment is provided by a GDS-50L type temperature controller with accuracy of ±0.1 °C. Natural frequencies of the tuning fork are obtained through the homemade software once every 10 °C from −20 °C to 60 °C first, and then from 60 °C to −20 °C. The experimental set-up and the software interface are shown in Figure 9.

**Figure 10.**
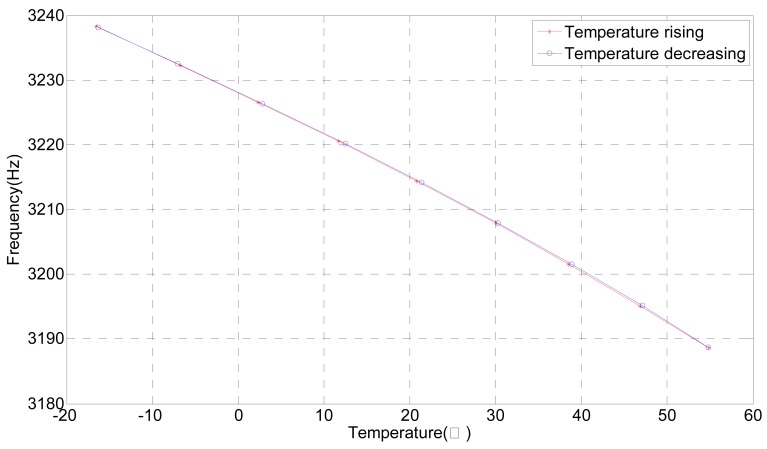
Natural frequency of the tuning fork changes with temperature.

Figure 10 shows that the natural frequency of the tuning fork changes with the temperature, thus, a temperature sensor is required in order to compensate for the changes in the elastic modulus of the tuning fork in real-time. The experiment result is identical with theoretical analysis provided above, which proves that the theoretical results are reliable.

## 5. Conclusions

In order to increase the precision of liquid density measurements, this paper designs a novel liquid density measurement sensor based on the resonance principle, and provides the calibration results of this sensor. Firstly, an approximate parameters model of the tuning fork is established according to the dynamic principle. The tuning fork structure of the sensor is also formed based on the optimization and improvements of further research. The impact of liquid density, position of the tuning fork, temperature and structural parameters on the natural frequency of the tuning fork are analyzed. Then the experimental results show that the repeatability error of the sensor is about 0.03% and the accuracy is about 0.4 kg/m^3^. In a word, the proposed density sensor equipped with a tuning fork as the resonant sensitive component based on the resonance theory is a high-precision instrument and can measure liquid density directly. The methods designed in this paper should also prove beneficial to research on other types of resonant sensors.

## Figures and Tables

**Table 1.** Material properties for stainless steel 316 L.

**Property**	**Young's modulus** *E* **(GPa)**	**Density** *ρ* **(kg/m^3^)**	**Thermal expansion** *α* **(10^−6^/°C)**	**Thermal conductivity** λ **(W/m/K)**	
Stainless steel 316 L	195	7,980	20∼100 °C	100 °C	300 °C

			16.0	15.1	18.4

**Table 2.** Experimental data at 20 °C.

**No.**	***ρ****_r_* **(kg/m^3^)**	***ρ***′ **(kg/m^3^)**	**Absolute error (kg/m^3^)**	**Relative error (%)**	**Repeatability (%)**
1	789.0	789.0	0	0	0.0073
	789.0	789.1	0.1	0.013	
	789.0	789.1	0.1	0.013	

2	834.0	833.8	−0.2	−0.024	
	834.1	833.9	−0.2	−0.024	0.0069
	834.0	833.9	−0.1	−0.012	

3	859.9	860.3	0.4	0.046	
	860.0	860.4	0.4	0.046	0.0067
	860.1	860.4	0.3	0.035	

4	998.1	998.2	0.1	0.010	
	998.0	998.0	0	0	0.015
	998.0	997.8	−0.2	−0.020	

5	1120.5	1120.7	0.2	0.018	
	1121.1	1120.8	−0.3	−0.027	0.032
	1121.0	1120.8	−0.2	−0.018	
